# Electromagnetic Wave-Absorbing and Bending Properties of Three-Dimensional Honeycomb Woven Composites

**DOI:** 10.3390/polym13091485

**Published:** 2021-05-05

**Authors:** Li-Hua Lyu, Wen-Di Liu, Bao-Zhong Sun

**Affiliations:** 1 Key Laboratory of High Performance Fibers & Products, Ministry of Education, Donghua University, Shanghai 201620, China; sunbz@dhu.edu.cn; 2 School of Textile and Material Engineering, Dalian Polytechnic University, Dalian 116034, China; 18108210003147@xy.dlpu.edu.cn

**Keywords:** 3-D honeycomb woven fabric, CB/CIP, mechanical property, EM wave-absorbing property

## Abstract

To avoid the delamination of the traditional three-dimensional (3-D) honeycomb electromagnetic (EM) absorbing composites and improving the defects of low mechanical properties, the 3-D honeycomb woven fabrics were woven on the ordinary loom by practical design. The fabrication of 3-D honeycomb woven EM absorbing composites was based on carbon black/carbonyl iron powder/basalt fiber/carbon fiber/epoxy resin (CB/CIP/BF/CF/EP) by the vacuum-assisted resin transfer molding (VARTM) process. A CB/CIP composite absorbent study showed that CB/CIP composite absorbent belongs to a magnetic loss type absorbent. Adding CB/CIP significantly improved the absorption performance of composite, increased the absorption peak and the effective absorption bandwidth (EAB), but the bending performance decreased. The normalization analysis results showed that when the thickness was 15 mm, the mechanical properties and EM wave-absorbing properties of the 3-D honeycomb woven composite were the best matches. The morphological characteristics and displacement load curves of the composite after fracture were analyzed. The bending failure modes were brittle fracture of the fiber bundle, matrix cracking, and typical shear failure. Despite the above failure mechanism, the 3-D honeycomb woven EM absorbing composites still has good integrity without delamination.

## 1. Introduction

The problem of electromagnetic (EM) interference and radiation has stimulated the research interest in EM absorbing composites with thin thickness, low density, broadband and strong absorption [1]. The research of EM absorbing composites has gradually turned to structural EM absorbing composites, which have the functions of load-bearing and a broadband EM energy absorbing capability. At present, the research of structural absorbing composites, such as foam EM absorbing composites and honeycomb EM absorbing composites, is being studied to overcome the disadvantages of traditional EM absorbing composites, such as high density, weak absorption and narrow bandwidth [2]. Compared with other structures, the honeycomb structure can not only be used as a part of the main load-bearing structure, but also as a carrier of EM absorbing composites and absorbing medium [3,4]. Li et al. [5] prepared carbon-coated honeycomb absorbing composites and analyzed in detail the effects of honeycomb aperture, honeycomb height, coating thickness, dielectric constant, and dielectric loss factor on EM absorption. Wang et al. [6] integrated shorted carbon fibers into honeycomb frames as a dual-functional material for radar absorption and structural reinforcement, which showed excellent EM absorption at frequencies ranging from 2–18 GHz. Sun et al. [7] used a honeycomb structure of aramid paper impregnated with EM absorbent as the core layer and quartz fiberboard as the skin to form a composite sandwich structure with EM absorption ability bonding. The experimental results showed that the minimum reflection loss (RLmin) was −29.5 dB, and the EAB was 13.1 GHz.

At present, honeycomb structure EM absorbing composites are mostly prepared by the lamination method, which has some disadvantages which were under high temperature and high humidity environments or alternating external forces, the honeycomb structure EM absorbing composites are easy to break [8,9,10]. Therefore, the traditional honeycomb structure EM absorbing composites have difficulty achieving load-bearing and a broadband EM energy absorbing capability.

However, three-dimensional (3-D) honeycomb woven composites are composites which are made of 3-D honeycomb woven fabric as reinforcement and resin or other polymers as the matrix. Three-dimensional honeycomb woven fabric is a kind of 3-D structure textile material. In the fabric structure of the textile material, the concept of vertical yarn was introduced along the thickness direction of the material, and the vertical yarn was used to connect the warp yarn and the weft yarn. There were fiber bundles and reinforcement structures through the thickness direction to enhance the integrity of the textile materials. Therefore, 3-D honeycomb woven composites have good integrity and structural stability and can solve the interlayer problems of traditional honeycomb structure EM absorbing composites [11]. Three-dimensional honeycomb woven fabrics have been applied in a number of practical applications, for example fibrous porous media [12]. Bayraktar et al. [13] prepared a 3-D honeycomb woven composite and carried out low-speed impact tests on fabric samples using a drop-weight impact tester. The experimental results showed that the energy absorbed by the honeycomb structure was more significant than that absorbed by the plate sample. The honeycomb woven fabric showed good mechanical properties. Lv et al. [14] prepared 3-D honeycomb structure composites using glass fiber filament yarn/BF filament yarn on the ordinary loom and tested their bending properties, providing a reference structural optimization design and performance analysis of 3-D honeycomb woven composites. Zahid et al. [15] wove three kinds of 3-D honeycomb woven fabrics with different honeycomb sizes, and the breaking strength and elongation at break of the 3-D honeycomb woven fabrics were measured. The effect of honeycomb size on the mechanical properties should be considered in the design and weaving of 3-D honeycomb woven fabrics.

Current studies have shown that 3-D honeycomb woven composites have good anti-delamination abilities. However, there was no research on the EM absorbing property of 3-D honeycomb woven composites. Therefore, the low-cost design and preparation of 3-D honeycomb woven EM absorbing composites with the integration of load-bearing and absorbing structures and functions can realize the collaborative design of mechanical properties and EM absorbing properties the composite.

Therefore, in this study, three different thicknesses (7.5 mm, 15 mm, 22.5 mm) of 3-D honeycomb fabrics with ordinary looms were woven through practical design. The CB/CIP/EP as the matrix and the 3-D honeycomb woven EM absorbing composites were prepared by the VARTM process. Then, the coaxial method was used to study the EM properties of CB/CIP. The 3-D honeycomb woven EM absorbing composite used the United States Naval Research Laboratory (NRL) arch method to test the RL on 2–18 GHz. Next, the bending properties were tested by a microcomputer-controlled electronic universal testing machine. Finally, it discussed particles and absorbent material thickness absorbing the composites’ performance and the influence of mechanical properties, and experimental results obtained from the failure modes. An effective method to coordinate the design of absorbing and mechanical properties was proposed.

## 2. Materials and Methods

### 2.1. Materials and Equipment

EP JC-02A and the solidification reagent JC-02B (Changshu Jiafa Chemical Co., Ltd., Changshu City, China). 800 tex CF filament SYT49 (Zhongfu Shenying Carbon Fiber Co., Ltd., Lianyungang City, China). 800 tex BF filament (Zhejiang Shijin Basalt Fiber Co., Ltd., Hangzhou City, China). Ordinary loom SGA 598 (Jiangyin Tong Yuan spinning machine Co., Ltd., Wuxi City, China). The universal testing machine TH-8102S (Suzhou Tuobo Machinery Equipment Co., Ltd., Suzhou City, China). 

### 2.2. Preform Design and Weaving

**Figure 1:**
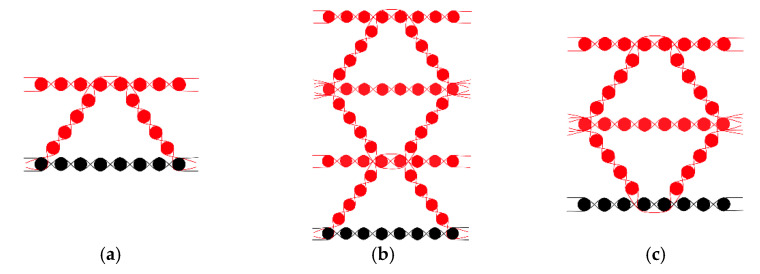
(**a**) Warp structural drawings of 7.5 mm 3D honeycomb woven fabric; (**b**) warp structural drawings of 15 mm 3D honeycomb woven fabric; (**c**) warp structural drawings of 22.5 mm 3D honeycomb woven fabric.

The warp structural drawings of 3-D honeycomb woven fabrics with three different layers are shown in Figure 1. As shown in Figure 1, the bottom warp and weft yarns were made entirely of 800 tex CF filament, and the rest of the warp and weft yarns were wholly made of 800 tex BF filament.

**Figure 2:**
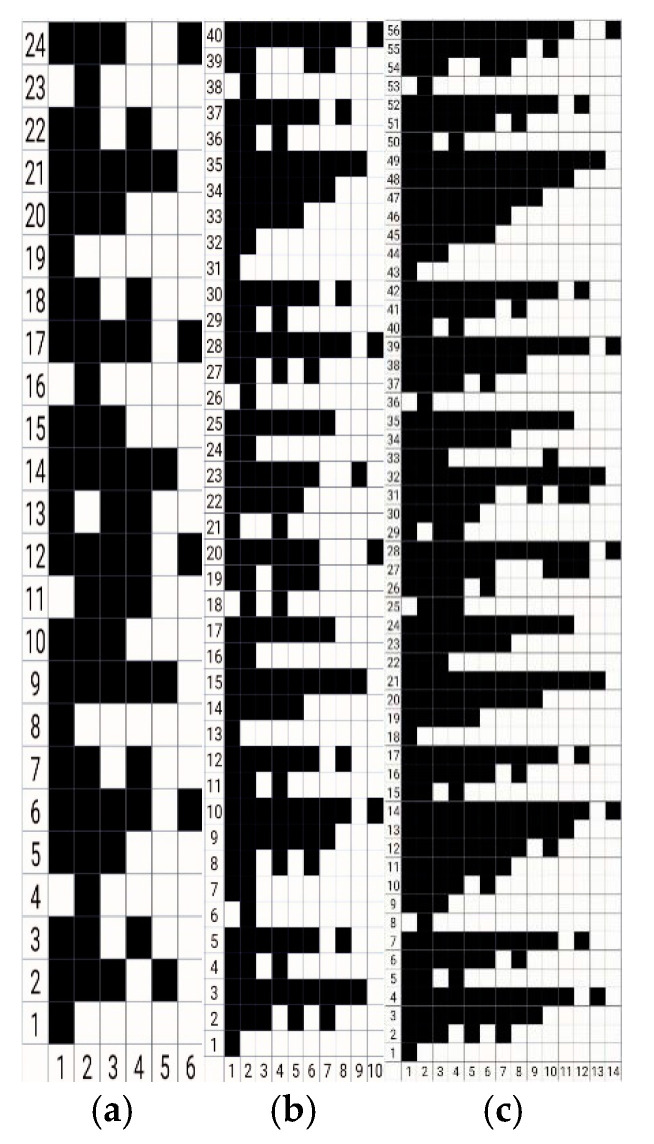
(**a**) Chain draft of 7.5 mm 3-D honeycomb woven fabric; (**b**) chain draft of 15 mm 3-D honeycomb woven fabric; (**c**) chain draft of 22.5 mm 3-D honeycomb woven fabric.

The chain drafts of 3-D honeycomb woven fabrics with three different thicknesses were drawn up according to the warp structural drawings, and they are shown in Figure 2. The weaving parameters of 3-D honeycomb woven fabrics are shown in Table 1.

### 2.3. Fabrication of the 3-D Honeycomb Woven EM Absorbing Composites

**Figure 3:**
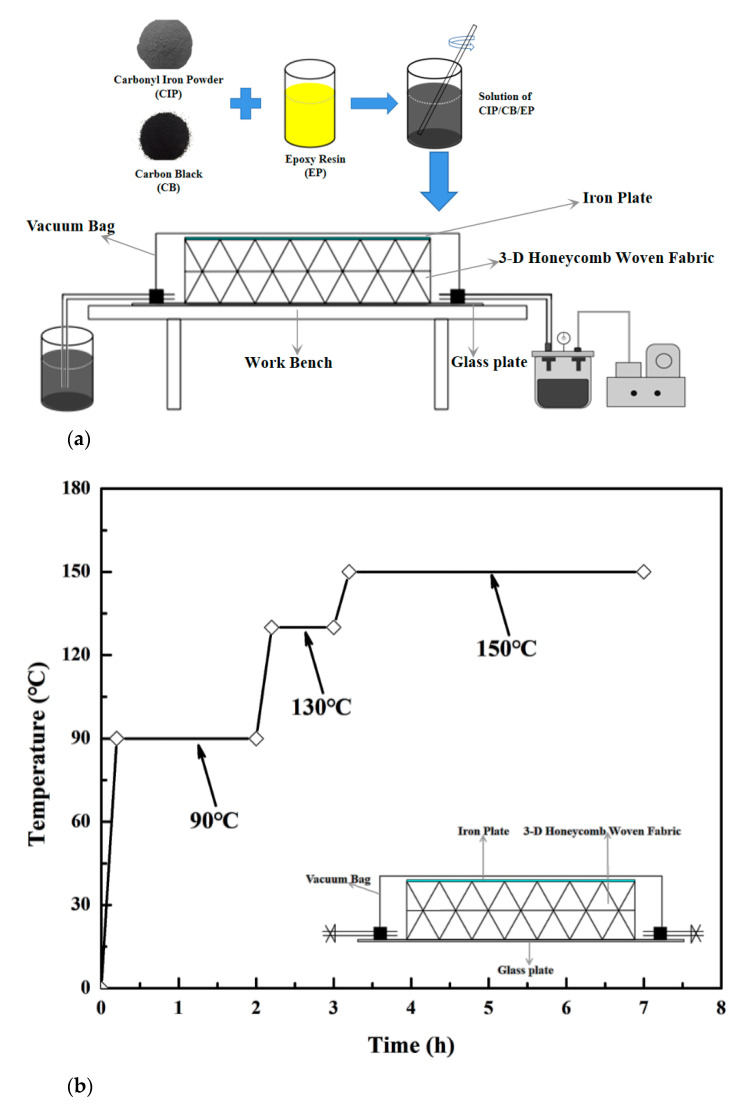
(**a**) Schematic diagram of VARTM process; (**b**) high-temperature curing process diagram of the 3-D honeycomb woven EM absorbing composite.

The ratio of the EP, CIP, solidification reagent, and CB was 4:4:3.2:0.03. VARTM manufactured the 3-D honeycomb woven EM absorbing composites. Figure 3a showed the schematic diagram of the VARTM process. The CB, CIP, and EP were mixed in a beaker and mechanically stirred until the CB/CIP could be uniformly dispersed in the EP without significant settling. After vacuum defoaming for 1 h in a vacuum drying oven at 60 °C, the CB/CIP/EP mixed solution without bubbles was obtained. The process involved placing the one-layer of 3-D honeycomb woven fabric (200 mm × 200 mm × 7.5 mm), the two-layer of 3-D honeycomb woven fabric (200 mm × 200 mm × 15 mm), and the three-layer of 3-D honeycomb woven fabric (200 mm × 200 mm × 22.5 mm) into their molds, closing the molds, and checking for leaks. Then, the resin solution was injected into the mold. The injection process was continued until a sufficient resin volume was seen in the resin trap to indicate that the mold had been completely filled with resin. The mold was isolated from the resin pot and the resin trap and then put into an air-circulating oven. The manufacturer-recommended cure cycle was employed: the first step of the cure cycle was 2 h at 90 °C, with the second step being 1 h at 130 °C, and the final step being 4 h at 150 °C, as shown in Figure 3b. The 3-D honeycomb woven EM absorbing composite without adding CB/CIP, with a thickness of 7.5 mm, was recorded as S0. The 3-D honeycomb woven EM absorbing composite with added CB/CIP, with a thickness of 7.5 mm, was recorded as S1. The 3-D honeycomb woven EM absorbing composite with added CB/CIP, with a thickness of 15 mm, was recorded as S2. The 3-D honeycomb woven EM absorbing composite with added CB/CIP, with a thickness of 22.5 mm, was recorded as S3.

### 2.4. Characterization Methods

**Figure 4:**
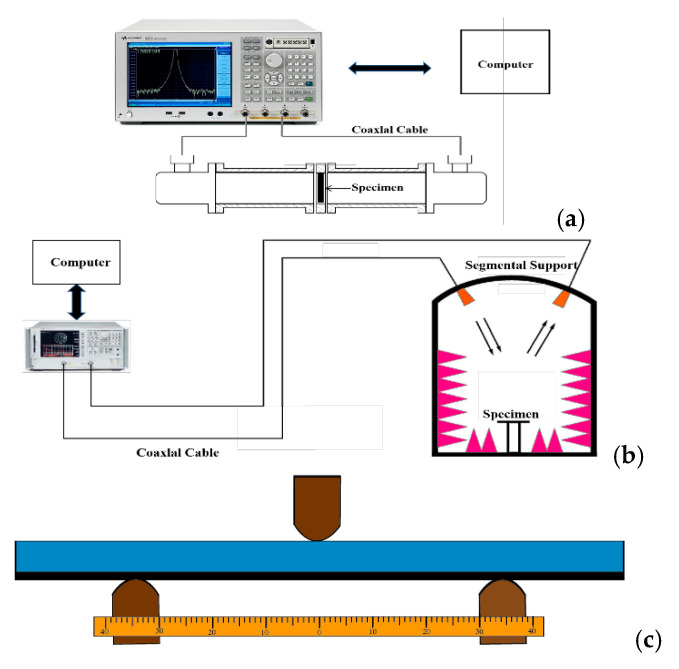
(**a**) Schematic diagram of the dielectric property test equipment; (**b**) schematic diagram of the EM reflection loss test equipment; (**c**) schematic diagram of the three-point bending test.

The EM parameters of the CB/CIP were tested by the coaxial method. The specimens were prepared by homogeneously mixing the CB/CIP with the paraffin in a mass ratio of 0.03:4:4; the mixture was pressed into a toroidal shape with an inner diameter of 3.04 mm and an outer diameter of 7 mm. The schematic of the experimental setup is shown in Figure 4a.

The morphology of the absorbent was observed with the scanning electron microscope JSM-7800F (JEOL Ltd., Guangzhou City, China) in the high-vacuum. The applied accelerating voltage was 15 kV and the working distance was 10 mm.

The United States NRL arch measurement system was used to evaluate the broadband EM characteristics of the 3-D honeycomb woven EM absorbing composites with different layers with a reference standard of GJB2038-2011; the schematic of the experimental setup is shown in Figure 4b.

Three-point bending with a universal testing machine followed GB/T 9341-2008. The loading rates were 10 mm/min for the bending test. The schematic of the experimental setup is shown in Figure 4c.

## 3. Results and Discussion

### 3.1. EM Properties

**Figure 5:**
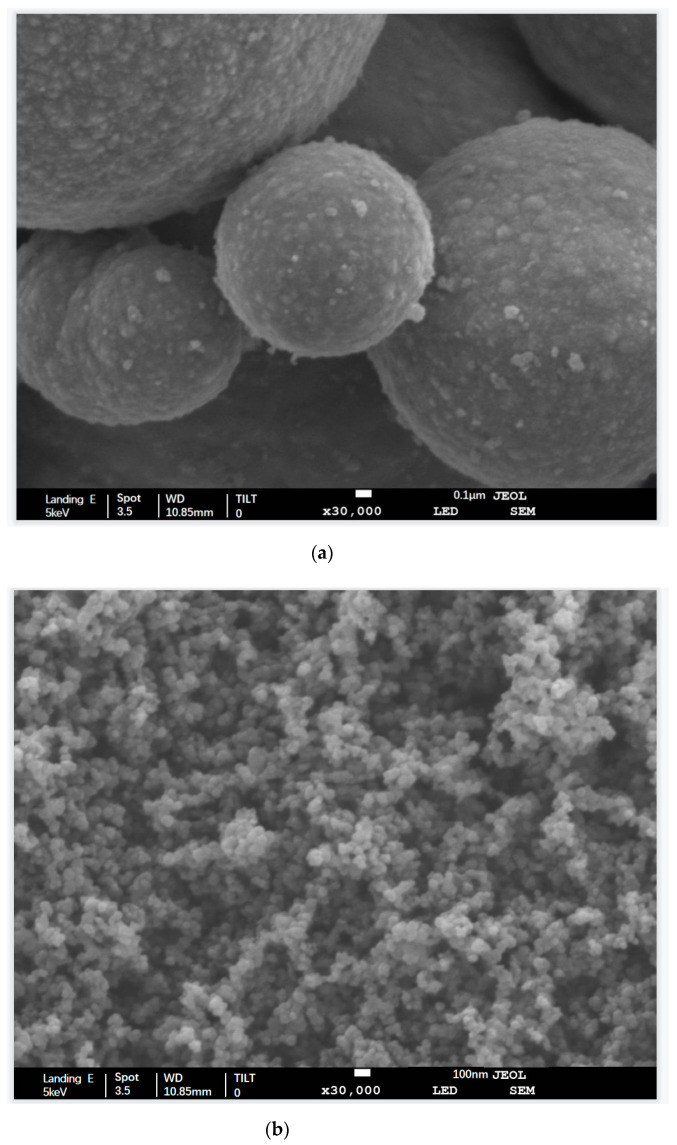
(**a**) Scanning electron microscope image of CIP; (**b**) scanning electron microscope image of CB.

The micro-structure of CIP and CB is shown in Figure 5a,b, respectively. As shown in Figure 5a, the microscopic morphology of CIP was spherical particles with a sphere structure, with an average particle size of about 3.5 μm. Such a spherical structure was also conducive to its uniform distribution in the matrix [16]. As shown in Figure 5b, the microscopic morphology of CB particles was approximately spherical, with a sphere size of about 20–30 nm. Under the electron microscope, the CB particles were agglomerated, caused by the high surface energy of nanoparticles. The agglomerated form made it easy for CB particles to form the conductive path in the matrix, which significantly improved CB’s conductivity.

**Figure 6:**
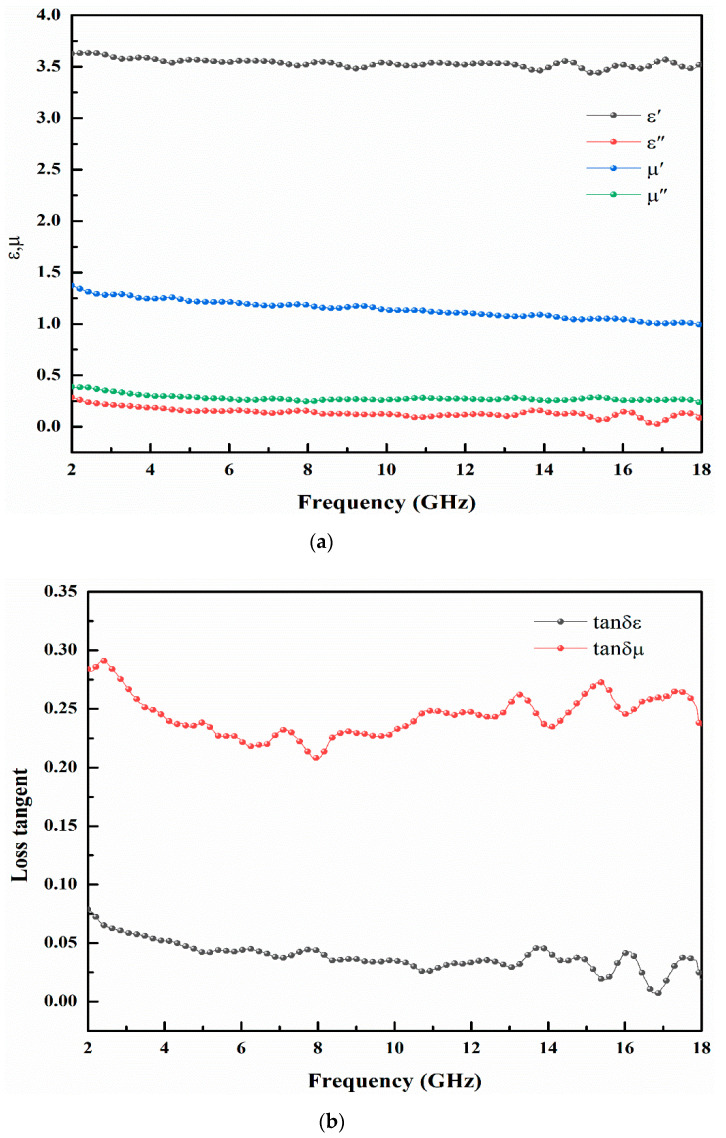
(**a**) EM parameters of CB/CIP particles; (**b**) loss tangent of CB/CIP particles.

The EM parameters of CB/CIP tested are shown in Figure 6a. From Figure 6a, it can be seen that the real part of the complex dielectric constant of CB/CIP fluctuates very little in the range of 2–18 GHz test frequency, generally distributed in the range of 3.5–3.75, and the imaginary part of the same dielectric constant ranges from 0 to 0.25, showing a weak dielectric loss performance. The real part μ′ and imaginary part μ″ of the complex permeability decreased gradually with the increase in frequency and were distributed in the range of 1–1.5 and 0.25–0.5, respectively. Figure 6b shows the curve of the loss tangent of CB/CIP. It can be seen that the dielectric loss tangent of CB/CIP tends to decrease in the measured range and eventually fluctuates around 0. These data indicated that CB/CIP has a weak dielectric loss performance. The magnetic loss tangent of CB/CIP decreased at first and then increased, which indicated that CB/CIP has good magnetic loss performance and is an excellent magnetic loss absorbent.

**Figure 7:**
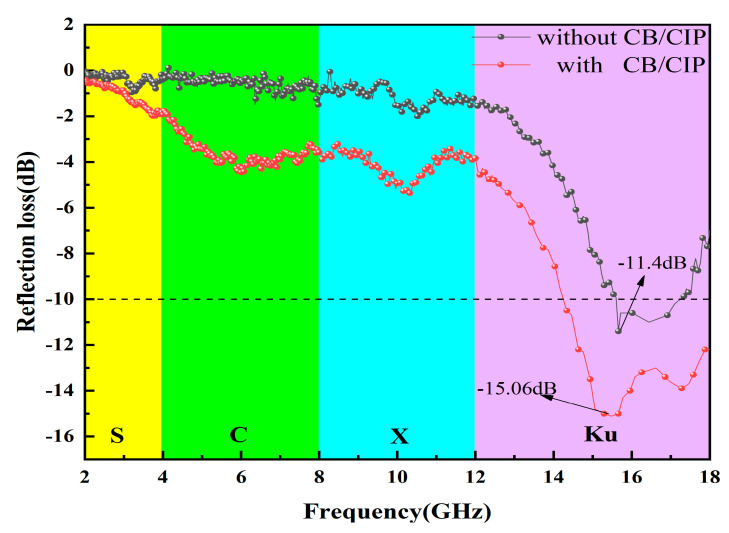
EM wave absorption performance of S0 and S1.

As shown in Figure 7, the S-band refers to the electromagnetic (EM) wave frequency range between 2 GHz and 4 GHz. The C-band refers to the EM wave segment with a frequency between 4 GHz and 8 GHz. The X-band refers to the EM wave segment with a frequency between 8 GHz and 12 GHz. The Ku-band refers to the EM wave segment with frequencies between 12 GHz and 18 GHz. The S0 reached the RL_min_ of −11.4 dB at a Ku-band (15.66 GHz). The S1 reached the RL_min_ of −15.06 dB at Ku-band (15.48 GHz). Therefore, we found that CB/CIP can improve the absorbing loss capacity of the whole composite.

The BF/EP (basalt fiber and epoxy resin) in the 3-D honeycomb woven EM absorbing composite were wave-permeable materials, which have no absorption ability to EM waves. The continuous CF layer mainly reflected the EM wave, and the EM wave that was not lost and was reflected twice after reaching the CF layer to improve the loss efficiency. CB/CIP, as an absorber of EM waves, was distributed in the EP matrix and mainly played the role of magnetic loss. CB/CIP can promote the formation of the absorbing network structure in the matrix, which improved the impedance matching and attenuation loss of the 3-D honeycomb woven EM absorbing composite, and the absorbing loss ability of the whole composite was enhanced. Compared to the two curves, we also found that with the addition of CB/CIP, the EM absorption intensity of the 3-D woven honeycomb EM absorbing composites increases, and the peak value of RL_min_ moved to the low-frequency direction. The RL curve showed the phenomenon that the absorption peak matches the frequency to move to the low frequency, which can be shown through the quarter wavelength theoretical explanation [17]:

(1) $$f_{m}=\frac{nc}{4d{\sqrt{\varepsilon_{r}\mu }}_{r}}(n=1,3,5\ldots )$$

*f_m_*: the absorption peaks match frequencies (GHz);

*c*: the speed of light (m/s);

*d*: the thickness of the 3-D woven honeycomb EM absorbing composite (mm);

$\varepsilon_{r}$: the relative dielectric constant;

$\mu_{r}$: the magnetic permeability.

It can be concluded that the matching frequency of the 3-D woven honeycomb EM absorbing composite was related to its thickness and electromagnetic parameters. According to the effective medium theory [18], it can be seen that the equivalent electromagnetic parameters of the mixture of absorbent and resin were related to the content of absorbent. When the thickness of the 3-D woven honeycomb EM absorbing composite was constant, the increase in CB/CIP content in Equation (1) led to the increase in equivalent EM parameters of absorbent honeycomb, and the corresponding matching frequency of absorption peak decreased; in the final step, the absorption peak moved to low frequency.

**Figure 8:**
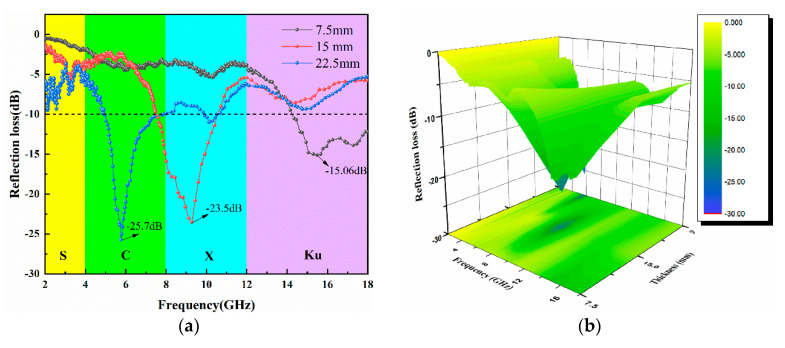

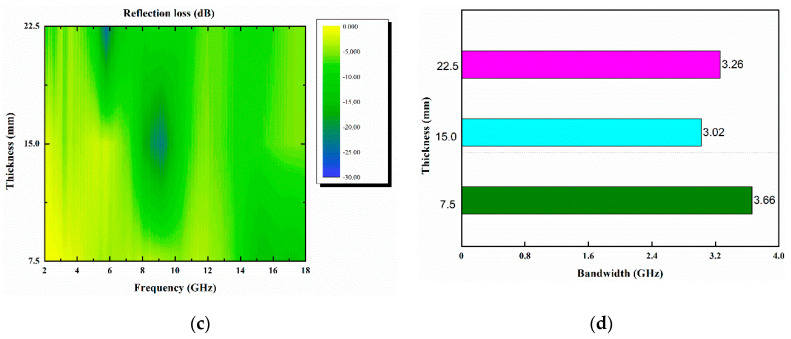
(**a**) RL curves of S1, S2, S3; (**b**) 3-D RL of S1, S2, S3; (**c**) two-dimensional plane RL of S1, S2, S3; (**d**) EAB of S1, S2, S3.

The RL curve of the 3-D honeycomb woven EM absorbing composites is shown in Figure 8. The RL_min_ of S1 was -15.06 dB (Figure 8a,b), and the EBA of the Ku–band was 3.66 GHz (Figure 8a,c,d), and the corresponding absorption band accounts for 61%. For S2, RL_min_ was -23.5 dB (Figure 8a,b). Although the EBA of the C–band (Figure 8a,c,d) was only 0.5 GHz and the corresponding absorption band occupies 12.5%, the EBA of the X-band was 2.52 GHz, and the corresponding absorption band occupies 63.8%. S3 obtained an excellent absorption intensity of -25.7 dB. In particular, the EAB of C–band (4–8 GHz) and X-band (8–12 GHz) low-frequency bands were 3.26 GHz and 0.19 GHz, respectively, and the corresponding absorption band occupancy rates were 81.5% and 19%, respectively, indicating that the C-band has excellent EM absorption performance. By comparing the three curves in Figure 8a, we also found that the EM absorption intensity of the 3-D woven honeycomb composites increases, and RL curves’ peak value moves to the low-frequency direction with the increase in thickness. The quarter wavelength theory can explain it. According to Equation (1), the thickness was inversely proportional to the absorbing frequency. That is to say, the thicker the thickness, the lower the absorbing frequency.

### 3.2. Bending Properties

**Figure 9:**
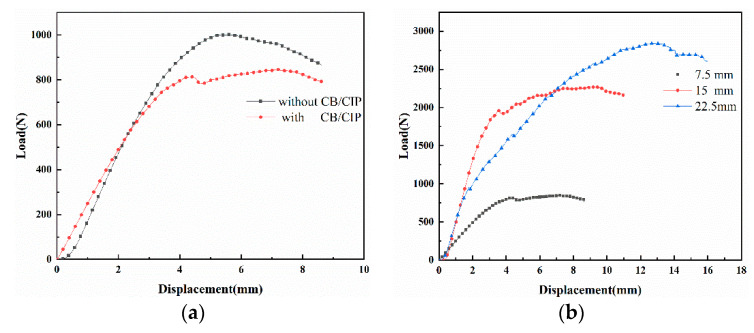
(**a**) Load–displacement curves of S0 and S1; (**b**) load–displacement curves of S1, S2 and S3.

The three-point bending load–displacement curves of the 3-D honeycomb woven EM absorbing composites are shown in Figure 9. The maximum bending load of S0 was 1002.03 N. The maximum bending load of S1 was 846.38 N. The maximum bending load of S2 was 2271.07 N. The maximum bending load of S3 was 2843.03 N. The reason for the maximum bending load decrease in S1 was that when the composites was subjected to bending load, CB/CIP agglomerated in the resin matrix, and a large number of microcracks gathered around the agglomerated particles, which could easily lead to macroscopic cracking caused by stress concentration. The maximum bending load increased with the thickness due to the more fiber bearing force. Therefore, the maximum bending load increased. Figure 9 shows that the curve was divided into three stages. In the initial loading stage, the material as a whole bears the bending load and performs well within the initial displacement, and the bending load–displacement curve also increases linearly. Therefore, the composite material has good linear elastic properties. With the increase in displacement, the composites bore more extrusion pressure. The shape variables were different between the fiber and the resin, the upper fiber bundle was subjected to the extrusion, while the lower fiber bundle was subjected to stretching so that the fiber in the composite material was pulled out and then destroyed the interface between the fiber and matrix. In this way, the modulus and flexural stiffness of the composite decreased, and the matrix cracked. Finally, the bending load–displacement curve showed a peak load and then began to decline. The load–displacement curve was convex on the whole.

The bending strength can be calculated according to the width, thickness, span, and displacement of the sample, and its equation is as follows:

(2) $$\sigma =\frac{3PL}{2bd^{2}}$$

**Figure 10:**
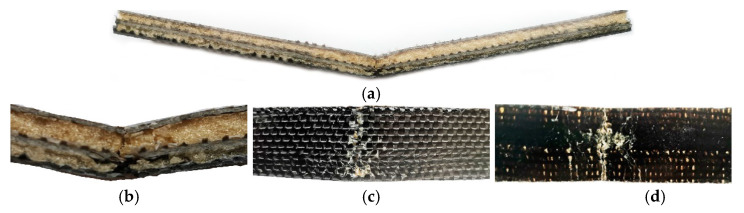
(**a**) Overall view of S2; (**b**) partial side view of S2; (**c**) partial bottom view of S2; (**d**) partial top view of S2.

σ: the bending strength (MPa);

*P*: the load (N);

*L*: the span (mm);

*b*: the width (mm);

*d*: the thickness (mm),

When the thickness was 7.5 mm, without CB/CIP, the bending strength was 106.88 MPa. When the thickness was 7.5 mm, with CB/CIP, the bending strength was 90.28 MPa. When the thickness was 15 mm, the bending strength was 121 MPa. When the thickness was 22.5 mm, the bending strength was 101.09 MPa. However, by comparing the load–displacement curve and bending strength of S0 and S1, it was found that the maximum load and bending strength of S1 were reduced. This may be due to the mutual attraction of the CB/CIP surface, resulting in the particle agglomeration phenomenon, resulting in the spatial barrier effect, and CB/CIP will also lead to the decrease in the crosslinking density of EP, resulting in the increase in defects, and the continuity of the EP matrix will be broken. As a result, the strength of the interface between the EP and the fibers weakens, eventually leading to direct cracking and failure of the composites at the CB/CIP aggregation sites. By comparing the bending strength of S1, S2, and S3, it was found that the bending strength of 3-D woven honeycomb EM absorbing composites increased first and then decreased with the increase in layers. This was mainly because, in the bending test, the upper BF was subjected to the action of compression force, the middle layer was subjected to the action of shear force, the lower CF was subjected to the action of tensile force. When the number of BF layers was two, the volume content of BF did not change much because the number of BF layers did not increase much. The bending strength of S2 was eventually increased. Subsequently, the number of layers of BF continued to increase, and the thickness of the 3-D woven honeycomb EM absorbing composites also increased. According to Equation (2), the bending strength was inversely proportional to the square of the thickness and positively proportional to the first power of the span. In contrast, the effect of thickness on bending strength was more obvious. Thus, S3 showed a decrease in bending strength. Additionally, with the increase in the number of BF layers, the volume content of BF gradually increased. Therefore, the effect of the strength of BF was also more obvious. As the strength of BF was lower than that of CF, the bending strength of S3 decreased. The results of the 3-D honeycomb woven EM absorbing composites with different thicknesses were similar. Thus, only the 3-D honeycomb woven EM absorbing composite with S2 was used as an example. Figure 10a–d shows the overall view, partial side view, partial bottom view and partial top view of S2, respectively. It can be seen from Figure 10 that the bending failure modes had a brittle fracture of the fiber bundle, cracking of the matrix, and a typical shear failure. However, the 3-D honeycomb woven EM absorbing composite had good integrity, and there was no delamination.

### 3.3. Mechanical Properties and EM Properties Match

It was necessary to evaluate the comprehensive properties of the composite materials, which met the requirements of the mechanical properties and met the requirements of the absorbing properties. Table 2 shows the mechanical properties and absorbing properties of the 3-D honeycomb woven EM absorbing composites.

To more intuitively represent the variation trend and amplitude of different thicknesses, mechanical properties, and EM absorbing properties and quickly match the optimal thickness, the concept of the normalized factor β was introduced [19]. Pure BF/CF/EP composite absorbing material without adding CB/CIP particles was regarded as the mechanical performance standard, and the minimum requirement of RL value −10 dB was regarded as the absorbing performance standard. The normalization factors of both were shown in Equations (3) and (4). The data in Table 3 were obtained after the normalization of the data in Table 2.

(3) $$\beta_{1}=\frac{\sigma_{f}-\sigma_{0}}{\sigma_{0}}(f=1,2,3)$$

(4) $$\beta_{2}=\frac{R_{f}-\left(-10\right)}{-10}(f=1,2,3)$$

$\beta_{1}$: Normalization factor of mechanical properties;

$\sigma_{f}$: Mechanical properties of S1, S2 and S3;

$\sigma_{0}$: Mechanical properties of S0;

$\beta_{2}$: Normalization factor of EM absorbing properties;

*R_f_*: EM absorbing properties of S1, S2 and S3.

**Table 3:** The mechanical properties and absorbing properties of normalized the 3-D honeycomb woven EM absorbing composites.

Specimen	Mechanical Properties	EM Properties	
	Bending Strength β	$\mathrm{RL}\min_{\;}\beta$	EAB (GHz)
S0	0	14%	0.3
S1	−15.53%	50.6%	3.66
S2	13.21%	135%	3.02
S3	−5.79%	157%	3.26

It can be seen from Table 3 that the bending strength of S1 and S3 decreased, so S2 conforms to the principle of matching the absorbing property and mechanical property, and this specimen met the requirements of both absorbing property and bearing capacity.

## 4. Conclusions

To meet the requirements of modern absorbing composites, which are the integration of load-bearing and EM wave absorption, and improve the low mechanical properties and overall performance of traditional honeycomb absorbing composites, the 3-D woven honeycomb EM absorbing composite was designed. To determine the effects of CB/CIP and thickness on the EM and mechanical properties of the 3-D honeycomb EM absorbing composites, the 3-D honeycomb EM absorbing composite without adding CB/CIP was designed. Three kinds of 3-D honeycomb EM absorbing composites with different thicknesses were prepared.

In the aspect of EM absorbing properties, the CB/CIP can promote the formation of the absorbing network structure in the matrix, which improved the impedance matching and attenuation loss of the 3-D honeycomb woven EM absorbing composite, so the absorbing loss ability of the whole composite was enhanced. When the thickness of the 3-D honeycomb EM absorbing composites was 15 mm and 22.5 mm, respectively, the composites’ absorptivity in the X-band and C-band was 63.8% and 81.5%, respectively. This was because with the increase in thickness, the fiber layer number was increased, and the hole was increased, the electromagnetic wave occurred in the material reflection, and the number of refractions was increased. Thereby, there was an increase in the electromagnetic waves absorbed. Besides, with CB/CIP and the increase in thickness, the RL peak of 3-D woven honeycomb EM absorbing composites moved to low frequency. This fit the quarter wavelength theory.

In terms of mechanical properties, the EP matrix’s continuity was broken easily due to the large amount of agglomeration of CB/CIP, and the strength between the resin and dimensional interface was weakened, thus reducing the mechanical properties of the 3-D honeycomb EM absorbing composites. Additionally, the results showed that the bending load of the 3-D honeycomb EM absorbing composites increased with the increase in the thickness of the composites, and the bending strength increased first and then decreased with the increase in the thickness of the composites. The 3-D honeycomb EM absorbing composites’ failure modes were mainly fiber bundle brittle fracture, matrix cracking, and typical shear failure. During the experiment, the 3-D honeycomb EM absorbing composite had no delamination and splitting phenomenon, and the overall performance was good.

After normalizing the mechanical and absorbing properties of the 3-D honeycomb EM absorbing composites, the optimal load/absorbing ratio was S2. The thickness was 15 mm, the maximum bending load was 2271.07 N, the bending strength was 121 MPa, the RL_min_ was −23.5 dB, and the EAB was 3.02 GHz; the composites satisfied with both load-bearing and EM wave absorption capacity.

This work provides a simple way to improve the mechanical properties and EM absorption capacity of structural EM absorbing composites with the 3-D woven honeycomb structure. However, we only investigated the EM absorbing property of the composites in regard to adding absorbent and different thicknesses, and we need to design further a 3-D woven honeycomb composite with different aperture sizes to achieve EM absorbing so that 3-D woven honeycomb composite can be used in weapon systems, such as fighters, warships, and missiles.

## Figures and Tables

**Table 1:** Weaving parameters of 3-D honeycomb woven fabrics.

Thickness (mm)	Linear Density/Tex	Layer Number of Yarns	Weaving Density (Yarn/10 cm)	
	Warp/Weft Yarns		Warp Density	Weft Density
7.5	800	1	180	95
15		2	300	
22.5		3	420	

**Table 2:** The mechanical properties and absorbing properties of the 3-D honeycomb woven EM absorbing composites.

Specimen	Mechanical Property	EM Properties	
	Bending Strength (MPa)	RL_min_ (dB)	EAB (GHz)
S0	106.88	−11.4	0.3
S1	90.28	−15.06	3.66
S2	121	−23.5	3.02
S3	101.09	−25.7	3.26

## Data Availability

The data presented in this study are available on request from the corresponding author.
